# The role of color and attention-to-color in mirror-symmetry perception

**DOI:** 10.1038/srep29287

**Published:** 2016-07-11

**Authors:** Elena Gheorghiu, Frederick A. A. Kingdom, Aaron Remkes, Hyung-Chul O. Li, Stéphane Rainville

**Affiliations:** 1 University of Stirling, Department of Psychology, Stirling, FK9 4LA, Scotland, United Kingdom; 2 McGill Vision Research, Department of Ophthalmology, McGill University, Montreal, Canada; 3 Kwangwoon University, Department of Industrial Psychology, Seoul, Korea; 4 VizirLabs Consulting, Chelsea, Quebec, Canada

## Abstract

The role of color in the visual perception of mirror-symmetry is controversial. Some reports support the existence of color-selective mirror-symmetry channels, others that mirror-symmetry perception is merely sensitive to color-correlations across the symmetry axis. Here we test between the two ideas. Stimuli consisted of colored Gaussian-blobs arranged either mirror-symmetrically or quasi-randomly. We used four arrangements: (1) ‘segregated’ – symmetric blobs were of one color, random blobs of the other color(s); (2) ‘random-segregated’ – as above but with the symmetric color randomly selected on each trial; (3) ‘non-segregated’ – symmetric blobs were of all colors in equal proportions, as were the random blobs; (4) ‘anti-symmetric’ – symmetric blobs were of opposite-color across the symmetry axis. We found: (a) near-chance levels for the anti-symmetric condition, suggesting that symmetry perception is sensitive to color-correlations across the symmetry axis; (b) similar performance for random-segregated and non-segregated conditions, giving no support to the idea that mirror-symmetry is color selective; (c) highest performance for the color-segregated condition, but only when the observer knew beforehand the symmetry color, suggesting that symmetry detection benefits from color-based attention. We conclude that mirror-symmetry detection mechanisms, while sensitive to color-correlations across the symmetry axis and subject to the benefits of attention-to-color, are not color selective.

Mirror-symmetry (from now on just ‘symmetry’) occurs when two halves of a pattern mirror each other. Symmetry is a ubiquitous feature in natural images, and is found in both biological and man-made objects. Psychophysical and brain imaging studies (fMRI) have shown that symmetry perception plays an important role in object recognition^1^,^2^, figure-ground segregation^3^,^4^ (e.g. symmetrical regions often tend to be seen as figure rather than ground), amodal completion^5^ and visual search^6^, and involves a widespread network of higher-tier extrastriate visual areas, such as V3A, V7, and LOC^7^,^8^. Thus, understanding symmetry perception is at the very heart of understanding perceptual organization^9^. In this communication we provide new psychophysical evidence concerning the role of color and attention-to-color in symmetry processing.

It is well-established that color (chromatic) contrast facilitates the detection of otherwise camouflaged objects^10^,^11^. It is therefore not surprising that symmetry - an important biological feature - can be detected in isoluminant stimuli, that is stimuli defined solely by chromatic contrast^12^. That is, the human visual system is sensitive to the colors and not just the positions of features in the perception of symmetry. Figure 1 provides a simple demonstration that we are sensitive to color in symmetry perception. On the left is shown a figure with perfect symmetry in terms of the colors as well as the positions of the elements: the symmetry is perceived effortlessly. On the right is a pattern with perfect positional symmetry, but the colors are mismatched across the symmetry axis: now the symmetry is hard to perceive.

Figure 1 however begs the question: are symmetry mechanisms color*-selective* as opposed to being merely color-*sensitive*. That is, does the visual system have positional grouping mechanisms that are gated by color, for example a mechanism that groups all the green elements in the pattern and derives a ‘green-symmetry’ signal, another mechanism that groups all the red elements in the pattern and derives a ‘red-symmetry’ signal, another the blue elements, another the yellow etc. Interestingly, the literature is equivocal on the issue of whether there exist such channels in human vision. Using non-isoluminant patterns consisting of sixteen squares arranged either in perfect symmetry or with some pairs mismatched in color, Morales and Pashler^13^ compared reaction times for detecting symmetry between patterns made of two versus four colors and found that responses to the four-color pattern were less accurate. They concluded that symmetry detection mechanisms were in themselves color-blind, and argued that symmetry in multi-color patterns could only be detected by switching attention from one color to the next and assessing individually the symmetry for each color. On the other hand, Wu and Chen^14^ recently reported that a noise mask maximally elevated thresholds for detecting symmetry when the noise and symmetry elements were of the same chromaticity, arguing that symmetry perception must therefore be selective for color. However, while Wu and Chen’s result shows that symmetry mechanisms are *sensitive* to color correlations, it does not follow that there exist color-selective symmetry channels as we have defined them here. In Wu and Chen’s^14^ experiments observers were able to determine the color of the symmetric pattern and selectively attend to it, enabling them to segregate the symmetry elements from the noise, facilitating symmetry detection.

In this communication, we re-examine the issue of whether symmetry mechanisms are color selective using a different approach to that employed by the aforementioned studies, an approach inspired by a paradigm used previously to examine whether global motion mechanisms are color selective^15^. The key methodological feature of our study is that in all conditions the stimuli and task are designed in such a way as to prevent the symmetry color becoming known to the observer, unless cued in advance. In our stimuli the symmetry color never ‘pops out’ by virtue of being the minority color (as in Wu and Chen’s stimuli^14^) which would otherwise allow attention to be drawn to it (see also^15^). We do nevertheless consider the situation in which observers are cued to the symmetry color. For completeness, we have extended the paradigm to luminance polarity (black versus white blobs), and consider whether attention-to-luminance-polarity plays a role in symmetry perception. We also examine the effect of the number of blobs (or blob density) and the presentation duration of the stimuli.

Our ‘with symmetry’ chromatic stimuli are illustrated in Fig. 2. All stimuli contain 96 isoluminant colored blobs, divided equally either into two (red, green), three (red, green, blue) or four (red, green, blue, yellow) colors. There were 50% positionally-symmetric blobs in the two-color conditions (Fig. 2a), 33% positionally-symmetric blobs in the three-color conditions (Fig. 2b), and 25% positionally-symmetric blobs in the four-color conditions (Fig. 2c), the remaining non-symmetric ‘distractor’ blobs being randomly-positioned and drawn equally from the remaining colors. For the achromatic condition, as in the two-color condition, there were 50% positionally-symmetric blobs, either dark, bright or both blobs, and 50% distractor blobs that were randomly-positioned and drawn equally from the remaining polarity/polarities. Note that for each number-of-colors condition, the amount of positional-symmetry is identical for the ‘segregated’, ‘non-segregated’ and ‘anti-symmetric’ color-arrangements, as can be seen by comparing the two-color conditions in the left, middle and right panels in Fig. 2a, in which all stimuli contain 50% positional-symmetry. The ‘without-symmetry’ comparison stimuli were composed of the same colors as the with-symmetry stimuli but with all elements randomly positioned.

In the ‘segregated’ conditions (Fig. 2, left panel), the symmetry pattern is of one color and the distractors are of the remaining colors. In the two-color segregated example in Fig. 2a, the symmetry blobs are in green and the distractor blobs in red; in the three-color example (Fig. 2b), the symmetry is in green and the distractors blue and red; in the four-color example (Fig. 2c), the symmetry is in green and distractors in blue, red and yellow. In the ‘non-segregated’ conditions (Fig. 2, middle panel), the symmetry pattern consists of equal numbers of all colors, as with the distractors. In the ‘anti-symmetric’ condition (Fig. 2, right panel) the blobs in the symmetry pattern are mirror-symmetric in terms of position but not chromaticity, that is the colors are oppositely paired, e.g. red with green, blue with red, green with blue, and so on.

The segregated condition in the left panel of Fig. 2 is actually two conditions. In one, from now on termed the ‘segregated’ condition, the color that is symmetric is the same during the entire experimental session. It was this condition that we used to test for the potentially beneficial effects of attention, since in this condition the observer would know on each trial which color carried the symmetry signal. In the ‘random-segregated’ condition on the other hand, the symmetric color is randomly selected on each trial from the available colors, so observers never knew which color contained the symmetry signal.

In order to examine whether the number of blobs in the stimulus affected symmetry detection, we collected data for all the aforementioned conditions using also 36 and 120 blobs, as illustrated in Fig. 3 for the three-color condition – note that the symmetry signal-to-noise ratio is identical (33%) in these patterns. In addition, in order to examine whether stimulus presentation duration affected symmetry detection, we collected data for all aforementioned conditions using stimuli made of 96 blobs presented for 1000 ms.

To re-cap: there are three number-of-colors conditions, namely two, three and four colors, and three number-of-dots conditions: 36, 96 and 120 dots. For each of the nine combinations of number-of-colors and number-of-dots, data was collected for four color-arrangements, namely segregated, random-segregated, non-segregated and anti-symmetric. In addition, there were two attention conditions that were applied to the 96 dot stimuli. In the ‘without-attention’ conditions observers were not instructed to attend to any particular color. In the ‘with-attention’ conditions observers were instructed to attend to just one color in the display. For the segregated condition, the with-attention condition meant attending to the color of the symmetry pattern, which remained the same throughout the session. For the other color arrangement conditions the with-attention color was ostensibly arbitrary from the standpoint of aiding detection, but was included for the sake of completeness.

Symmetry detection was measured using a two-interval-forced-choice (2IFC) procedure. On each trial a ‘with symmetry’ and a ‘without-symmetry’ stimulus was presented for 500 ms with an inter-stimulus interval (ISI) of 500 ms. We measured proportion correct responses across trials. There was therefore just one value of proportion correct for each condition (for each observer) and it was found that this value in every condition lay between 50% and 100% correct, i.e. no condition was subject to a floor or ceiling effect. Nevertheless some might question why we did not collect whole psychometric functions of proportion correct as a function of some independent variable that controlled performance, for example the amount of positional jitter added to the symmetry dots, or proportion of dots containing the symmetry signal. Our method embodied constraints imposed on our stimuli in order to answer the specific questions posed in this study. For example, if we had manipulated the proportion of dots containing the symmetry signal in order to change the performance level we would have compromised the requirement to eliminate all cues as to what color contained the symmetry signal. Consider for example the two-color random-segregated condition. By manipulating the proportion of dots carrying the symmetry signal, one would necessarily co-vary the proportion of dots that were of one of the two colors. Thus, there would in all except the 50% symmetry condition be a minority color which would tend to ‘pop-out’, grabbing the attention of the observer. On half the trials this minority color would carry the symmetry signal (in one of the two intervals), but on the other half it would not (because the color containing the symmetry signal was randomized on each trial), so it is possible that performance would be compromised, one way or the other, if observers tended to attend to the pop-out color. In all our stimuli the numbers of each color were always identical in order to eliminate any such pop-out effects.

Let us now consider how the four different color arrangement conditions can be used to answer the following questions: 1. Is symmetry detection sensitive to color-correlations? 2. Are there color-selective symmetry channels? 3. Does attention-to-color facilitate symmetry detection? To answer the first question we compare results between the non-segregated and anti-symmetric conditions: if symmetry detection is sensitive to color correlations we would expect the anti-symmetric condition to fare worse, in line with the demonstration in Fig. 1. To test for the presence of color-selective symmetry channels (second question), we compare performance from the random-segregated with non-segregated conditions. If there are color-selective symmetry channels, we would expect performance to be better for the random-segregated condition. Why? In the random-segregated condition the symmetry signal is carried by just one color, with no distractors of that color. In the non-segregated condition on the other hand, the symmetry signal is carried equally by all colors and the distractors also by all colors. On the assumption that if color-selective symmetry channels exist the independent signals from them would be combined by probability summation, then we should expect performance in the random-segregated conditions to be better than that for the non-segregated conditions. This is so because in the random-segregated condition the local symmetry signals would be additively combined into a single color-selective symmetry channel, producing a relatively large symmetry signal in that color channel and zero symmetry signal in the other color channels. In the non-segregated condition on the other hand, there would be symmetry information in all channels but the information in each channel would be much weaker by virtue of the information from the equal numbers of distractors. Probability summation across channels would result in an overall stronger signal in the random-segregated compared to non-segregated condition^16^. If there are no color-selective symmetry channels, then all color-symmetry signals will be pooled into one single channel. Based on the actual experimental data for the random-segregated condition we have derived predictions for the non-segregated condition based on the probability summation of independent color-symmetry channels within the framework of signal-detection theory.

Finally, for the third question we compare the ‘with-attention’ and ‘without-attention’ segregated conditions, with the prediction that if attention is useful then performance will be superior in the former condition.

## Results

The results for the conditions in which the observers did not know the symmetry color or were not required to attend to a particular color are shown in Fig. 4. The figure shows results for each number of colors. The data are for three observers and the average across five observers is also shown. Proportion correct responses for the non-segregated and anti-symmetric conditions are shown in Fig. 4a and for the non-segregated and random-segregated conditions in Fig. 4b. The corresponding results for the luminance-polarity (bright and dark blobs) are shown in Fig. 5a–b. Consider how these results address our three main questions.

### Is symmetry detection sensitive to color-correlations?

One can see from Figs 4a and 5a that performance in the anti-symmetric conditions is lower than in the non-segregated condition. Paired-sample t-tests on the logit transformed data show that the differences are statistically significant at 0.05 level for all conditions: t(4) = 9.984, p = 0.0006 for achromatic patterns; t(4) = 5.111, p = 0.007 for two-color patterns; t(3) = 4.676, p = 0.018 for three-color patterns and t(3) = 5.217, p = 0.013 for four-color patterns. The lower performance with anti-symmetric patterns confirms that symmetry perception is sensitive to color and luminance-polarity correlations across the symmetry axis.

### Are there color-selective symmetry channels?

Consider now Fig. 4b. There is no difference between the random-segregated and non-segregated conditions with either the two, three or four-color patterns, and similarly for the achromatic patterns in Fig. 5b. A paired-samples t-test analysis on the logit transformed data confirmed that performance in the random-segregated and non-segregated conditions was not statistically significant: t(4) = 1.435, p = 0.225 for achromatic patterns; t(4) = 0.851, p = 0.443 for two-color patterns; t(3) = 1.168, p = 0.327 for three-color patterns and t(3) = 3.063, p = 0.055 for four-color patterns).

In order to test directly whether the relationship between the random-segregated and non-segregated data supports the presence of independent color-selective symmetry channels, we used a signal-detection-theory analysis of probability summation^16^,^17^,^18^ using the formulae in Kingdom *et al*.^16^ as implemented in the Palamedes toolbox^19^. We used the random-segregated data (in which all the symmetry signals were carried by a single color, with no distractors of that color) to derive the predicted proportion correct data for the non-segregated conditions (in which there were equal numbers of symmetry and distractors in each color) assuming probability summation of independent color-symmetry channels.

**Figure 2:**
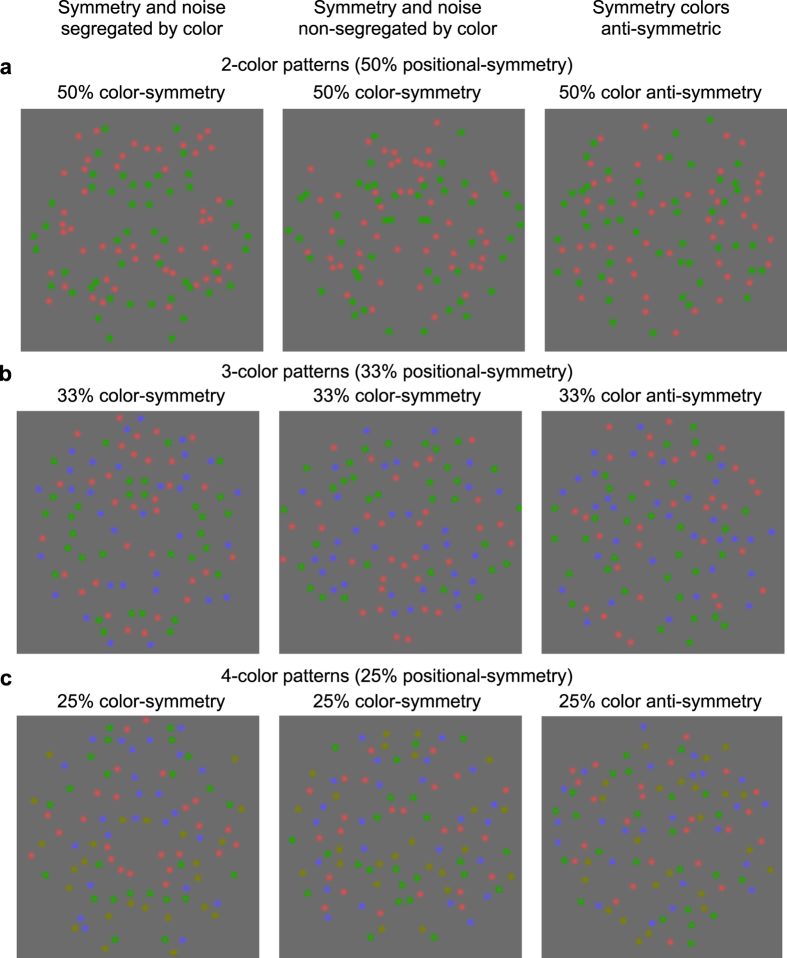
Example with-symmetry chromatic patterns used in the experiments. The patterns consisted of (**a**) two (red, green), (**b**) three (red, green, blue) and (**c**) four (red, green, blue, yellow) colors with respectively 50%, 33% and 25% blobs arranged symmetrically, the remaining blobs being randomly positioned and drawn equally from the remaining colors. For each number-of-colors condition, the left-to-right panels show the three types of color arrangement: (i) the ‘segregated’ condition (left panel) in which the symmetry pattern is of one color and the distractors are of the remaining colors. The example in the two-color case (**a**) shows the symmetry blobs in green and the distractor blobs in red; in the three-color case (**b**), the example shows symmetry in green and distractors in blue and red, and in the four color case (**c**), the example shows symmetry in green and distractors in blue, red and yellow. (ii) the ‘non-segregated’ condition (middle panel), in which the symmetry pattern consists of equal numbers of all colors, as with the distractors. (iii) the ‘anti-symmetric’ condition (right panel) in which the blobs are mirror-symmetric in terms of position but not chromaticity, that is the colors are oppositely paired, e.g. red with green, blue with red, green with blue, and so on.

To understand the principle behind the probability summation model consider the two-color case (Fig. 2a). In the two-color, no-attention random-segregated condition there were 48 symmetry dots of one color (red or green) and 48 distractor dots of the other color (left panel Fig. 2a), but the observer did not know on each trial which color contained the symmetry. In the two-color non-segregated condition (middle panel Fig. 2a) there were 24 symmetric and 24 distractor dots of one color (e.g. green), and 24 symmetric and 24 distractor dots of the other color (e.g. red). We assume that (1) if color-symmetry channels exist they will contribute their independent signals to detection by probability summation, (2) the strength of the symmetry signal in each color-symmetry channel is proportional to the proportion of dots of that color that are symmetric, and (3) the observer monitors the information in all color-symmetry channels for which there are colors in the stimulus. From these three assumptions, performance in the random-segregated condition results from the probability summation of one, full-strength (48 green symmetry blobs) and one, zero-strength (0 red symmetry blobs) signal, and in the non-segregated condition from the probability summation of two, half-strength (24 red and 24 green symmetry blobs) signals. For the three-color condition, the same principle applies: performance in the random-segregated condition results from the probability summation of one full-strength and two zero-strength (0 red and 0 blue blobs) signals, and in the non-segregated condition from the probability summation of three, one-third-strength signals. And so on for the four-color case.

To test the model we used the equation for the probability summation of equal intensity stimuli given in Kingdom *et al*.^16^. This equation is implemented by two routines in the Palamedes Toolbox^19^: PAL_SDT_PS_PCtoSL, which converts proportion correct to stimulus intensity, and its inverse PAL_SDT_PS_SLtoPC, which converts stimulus intensity to proportion correct. The first of these, PAL_SDT_PS_PCtoSL takes six arguments: proportion correct (*PC*), a scaling factor *g* for converting signal intensity to the signal-detection-theory measure *d*’ (“d-prime”), *τ* the exponent on the transducer function (for our situation the function that relates perceived to physical symmetry), *M* the number of alternatives in the forced-choice task, *Q* the number of monitored channels, and *n* the number of channels that are activated, i.e. that contain a stimulus. The routine outputs a value of stimulus intensity, or stimulus level *SL*, which for our purposes corresponds to the probability-summed strength of the symmetry signal. Beginning with the random-segregated case, we input the measured *PC* to the routine and obtain the output value of *SL*. For the other input arguments we set *g* (arbitrarily) to unity, *τ* to unity, Μ to 2 (2IFC task), *Q* to the number of monitored channels, in this case the number of colors/luminance-polarities (2, 3 or 4) and *n* to 1. The value for *n* was set to 1 because in the random-segregated condition only one color carried the symmetry signal. In order to make the *PC* prediction for the non-segregated condition we used the second routine PAL_SDT_PS_SLtoPC. The value of *SL* from the first routine was divided by the number of colors and input to the routine, and *n* was set to the number of colors. All other input parameters were the same as before. The details of the equations are described in *Appendix A* (*see* Supplementary information).

The average model predictions across observers for the proportion correct in the non-segregated conditions are shown in Fig. 6 for each number-of-colors condition (Fig. 6a) and for the luminance condition (Fig. 6b). For comparison we re-plotted the experimental data for the random-segregated and non-segregated conditions. As Fig. 6 shows, the predicted proportion correct based on the probability summation model is in nearly all cases lower than the measured proportion correct. Although the differences between the predicted and actual non-segregated data are small, at issue is whether the predicted data is *significantly* lower than the actual data. We tested this using one-tailed paired-sample t-tests on the logit-transformed data. We found that the differences between predicted and actual data were statistically significant for the achromatic patterns (t(4) = 2.705, p = 0.026), the two-color patterns (t(4) = 2.965, p = 0.0207), the three-color patterns (t(3) = 3.237 p = 0.024) but not for the four-color patterns (t(3) = 0.834, p = 0.232), for which the predicted and actual differences are very small to begin with. This modeling exercise shows that the probability summation model predicts results that are for the most part significantly lower than those actually found, so our data do not support the idea of independent color-selective symmetry channels that combine their signals by probability summation.

### Does stimulus density (i.e. number of blobs) affect symmetry detection?

The average across-observers results obtained with different numbers of blobs (36, 96 and 120) are shown in Fig. 7a for the non-segregated and anti-symmetric conditions, and in Fig. 7b for the non-segregated and random-segregated conditions. The corresponding results for the luminance-polarity (bright and dark blobs) are shown in Fig. 7c,d. The individual observers data (three observers) for all these conditions are shown in Appendix B (*see* Supplementary Information). Proportion correct results are little affected by the number of blobs (compare left, right and middle panel), suggesting that the limiting performance factor in these stimuli is not the number of blobs but the signal-to-noise ratios. The results in Fig. 7 show a similar pattern as those in Figs 4 and 5 indicating that symmetry perception is sensitive to color (Fig. 7a) and luminance-polarity (Fig. 7c) correlations across the symmetry axis but not selective to color/luminance polarity (Fig. 7b,d).

### Does stimulus presentation duration affect symmetry detection?

Figure 8 presents results for both 0.5 and 1 sec durations. Proportion correct results (average across two observers) are shown in Fig. 8a for the non-segregated (light green symbols) and anti-symmetric (light magenta symbols) conditions and in Fig. 8b for the non-segregated and random-segregated (light grey symbols) conditions. The corresponding results for the luminance-polarity are shown in Fig. 8c,d. For comparison we also show the results obtained with the 500 ms presentation duration (darker symbols). The individual observers data (two observers) for all conditions are shown in Appendix C (*see* Supplementary Information). These results show that doubling the presentation duration to 1000 ms produces only a modest increase in performance (compare light and dark magenta/green/black symbols), that is, ~0.75 proportion correct for the two-color and 0.85 proportion correct for the achromatic conditions. On average, across all participants and stimulus conditions, the increase in proportion correct responses for the 1000 ms stimulus presentation was ~0.1 for the two color and achromatic conditions, ~0.05 for the three-color and 0.01 for the four-color conditions.

### Does attention-to-color facilitate symmetry detection?

Relevant results are shown in Fig. 9, for 96 blob stimuli. Compare the chromatic (Fig. 9a) and luminance-polarity (Fig. 9b) results for the no-attention (gray symbols) with the with-attention (blue symbols) conditions. One can see that performance is higher for the with-attention compared to no-attention conditions, for both chromatic and achromatic patterns. Paired-sample t-tests on the logit transformed data showed that the differences between the two conditions are statistically significant for all chromatic and achromatic patterns: t(4) = 7.358, p = 0.001 for achromatic patterns; t(4) = 2.808, p = 0.048 for two-color patterns; t(3) = 3.97, p = 0.028 for three-color patterns and t(3) = 3.325, p = 0.044 for four-color patterns. On average, across all participants and number of color conditions, the increase in performance due to attention was about 13.3%. Thus, our results indicate that attention-to-color/luminance-polarity plays a role in mirror-symmetry detection.

In addition, because the with-attention conditions were run after the no-attention conditions we wanted to know if there were any differences in performance due to practice effects for the random-segregated, non-segregated and anti-symmetric patterns. Figure 10 shows the average across-observers data for the random-segregated, non-segregated and anti-symmetric chromatic (Fig. 10a–c) and luminance (Fig. 10d) conditions obtained in the ‘with-attention’ (blue symbols) and ‘no-attention’ (gray symbols) conditions. Similar performance was found in the ‘no-attention’ and ‘with-attention’ situation for each stimulus condition, suggesting no practice effects. All the paired-sample t-tests showed that the differences between the ‘no-attention’ and ‘with-attention’ conditions were not statistically significant (p > 0.05).

## Discussion

Using multi-color patterns made of Gaussian blobs we examined the role of color/luminance-polarity and attention-to-color/luminance-polarity in detecting mirror-symmetry. Our results indicate: (a) superior performance for the non-segregated compared to anti-symmetric conditions, confirming that symmetry detection is sensitive to color correlations across the symmetry axis; (b) no significant superiority in performance for the random-segregated compared to non-segregated conditions, giving no support to the idea that there exist color-selective symmetry channels; (c) highest performance in the attention-to-segregated condition, suggesting that symmetry perception can benefit from color-based attention; (d) comparable performance across number of blobs suggesting that the limiting performance factor in these stimuli is not the number of blobs (or density of blobs) but rather the signal-to-noise ratio (i.e. 50% for the two-color, 33% for the three-color and 25% for the four-color conditions), and (e) doubling the presentation duration produces only a modest increase in proportion correct responses in these stimuli (i.e. ~0.1 increase).

Our results explain and unify seemingly divergent findings from a number of studies^13^,^14^ exploring the role of color in symmetry detection. They also complement other findings on the role of low-level features, such as spatial frequency^20^ in symmetry detection. Together these studies suggest that while symmetry mechanisms are sensitive to across-the-symmetry-midline correlations in low-level features such as color, luminance-polarity and spatial frequency, it is not the case that the visual system has separate channels for computing the overall amount of symmetry across the elements with, say, a particular color, luminance-polarity or spatial-frequency.

Some readers might wonder why in Fig. 2a, the symmetry in the segregated condition (left panel) is more easily perceived than the symmetry in the non-segregated condition (middle panel), an effect absent in the experimental data shown in Fig. 4b, which compares random-segregated and non-segregated conditions. The likely reason is that the demonstration in Fig. 2a allows the reader the time to attend to one color at a time and, once the color that carries the symmetry signal is established, the symmetry color ‘stands out’. In fact this observation reinforces our finding that when one color carries the symmetry signal, priori knowledge of the color facilitates symmetry perception (compare blue and dark gray symbols in Fig. 9).

We found that unless observers were pre-cued as to which color was symmetric (such that they could selectively attend to it), they performed no better than in either the random-segregated or non-segregated conditions. This finding goes against the idea that the human visual system automatically groups elements of a given color into a color-specific symmetry channel, in keeping with the previous results and conclusions of Morales and Pashler^13^. With regard to the masking results of Wu and Chen^14^, in which symmetry detection improved when symmetry and noise elements were different in color, one may conjecture that this was a result of selective attention to the symmetry color (since participants knew beforehand the color of the symmetric target, and the fact that the numbers of symmetry and noise blobs were unequal (see their Fig. 1b), showing 14 symmetric and 18 noise dots, resulting in a difference of about 12.5% between signal and noise), resulting in a modest ‘pop-out’ effect of the symmetry color.

Our results complement a number of previous findings exploring the role of color vision in texture-processing^21^,^22^ and in other types of figure-ground relationship, such as the detection of global motion^15^,^23^,^24^,^25^,^26^,^27^,^28^,^29^, and stereoscopic targets embedded in multiple-depth distractors^30^. All these studies failed to find evidence for color-specific channels mediating the particular motion/form/depth task. Analogous findings have also been found in regard to the role of luminance polarity in global motion processing^31^. Moreover those of the above studies that also explored the role of attention-to-color in global motion processing reached an identical conclusion to us. Croner and Albright^28^,^29^ were the first to explore the effect of segregating target and distractors by color in global motion, and suggested that one explanation for the advantage in the segregated condition was that subjects were able to selectively attend to the target colour. Li and Kingdom^15^,^30^ went on to directly test the attention-to-color idea using a global motion stimulus in which all pre-attentive cues as to the target color were removed, and found that global motion thresholds were no better when target and distractors differed in color. However, when subjects were cued as to the target color, performance increased. Snowden and Edmunds^27^ found that the advantage conferred by having signal and distractor elements segregated by color disappeared when the stimuli were viewed peripherally, and suggested that the reason for this was the inherent difficulty in attending to individual element colors in the periphery. Finally, Lu and Sperling^23^ showed that alternating-feature stimuli produce apparent motion only when the observers selectively attended to one of the features embedded in the stimulus.

The idea of feature-based attention, i.e. attention to a type of feature rather than to a visual location, is central to our understanding of the way attention operates in vision (see review by Carrasco^32^). Feature-based attention can be considered as a type of filter, selecting the relevant information while discarding the irrelevant. Feature-based attention thus enhances the signal-to-noise ratio of the task in question.

Our observers experienced great difficulty perceiving symmetry in the anti-symmetric patterns (see right panels in Fig. 2). This may seem surprising given previous studies using achromatic dots^33^, Gaussian blobs^34^,^35^,^36^ and center-surround elements of opposite polarity^37^,^38^, where at least at low-to-medium densities performance for symmetric and anti-symmetric patterns was similar. For example, Wenderoth^33^ found that 100% symmetric, 100% anti-symmetric and 50% symmetric (half of the dots were of same-polarity and half of opposite polarity) elicited similar performance at around 80% correct detection. This performance level is similar to that obtained in our study with achromatic symmetric-patterns (see Figs 5 and 7c,d). Wenderoth^33^ concluded that there was no relationship between symmetry detection and the degree of luminance-polarity correlation across the symmetry axis. The results of these aforementioned studies using anti-symmetric stimuli give credence to the idea that second-order channels, specifically those agnostic to luminance polarity, play a role in symmetry detection^35^,^37^,^38^. The exceptions to these findings were the results obtained with very dense dot displays^35^,^37^,^38^,^39^ made of large elements or containing different spatial frequencies^39^ for which the anti-symmetrical arrangement produced, like here, poor performance. However, Mancini *et al*.^39^ suggested that the equal sensitivity to symmetry and anti-symmetry found in previous studies^33^,^34^,^35^,^36^ might not be due to the fact that the stimuli elicited similar responses from second-order channels^37^,^38^,^40^,^41^. Instead, these authors suggest that sensitivity to symmetry arises from quasi-linear channels whereas sensitivity to anti-symmetry arises from attentional mechanisms that operate only in sparse displays. Thus, Mancini *et al*.^39^ suggest that anti-symmetry in achromatic patterns is only detected under conditions favorable to selective attention that registers the positional symmetry of individual dots irrespective of their luminance-polarity. However, this type of attentional resource is different from the attention-to-color/luminance-polarity used in our study. In our multi-colored patterns, attention was directed to just one of the colors, so one would not expect attention to benefit perfomance with our anti-symmetric stimuli, on any grounds.

Our results show that doubling the presentation duration to 1000 ms produces only a modest increase of about 10% correct detections for the two-color patterns. This might seem surprising given that 1000 ms is long enough to allow eye movements across the pattern to enable observers to compare symmetry pairs across colors or luminance polarities. However, Morales and Pashler^13^ measured reaction times for detecting symmetry in non-isoluminant patterns (checkerboard patterns) made of two and fours colors. These authors found that mean reaction time responses to four-color patterns were slower (~2000 ms) and less accurate than with two-color (~1200 ms) patterns and that responses to *symmetric* patterns were slower (~1800 ms) than responses to asymmetrical ones (~1400 ms), suggesting that symmetry detection cannot be done simultaneously by comparing different colors across the symmetry axis. Our results for the presentation duration experiment are also consistent with Morales and Pashler^13^ findings.

## Methods

### Participants

Six observers participated in the experiments: the first author and five subjects who were naive with regard to the experimental aims. Three observers (EG, RA, CM) took part in all stimulus conditions, one observer (JS) in the two- and three-color conditions and two observers (DW, SG) in the two-color conditions only. For the number of blobs experiment three observers (EG, RA, CM) took part in all stimulus conditions and for the stimulus presentation duration experiment only two observers (EG and RA) participated. All observers had normal or corrected-to-normal visual acuity and normal color vision. Observers gave their written informed consent prior to participating in this study and were treated in accordance with the Declaration of Helsinki. The research protocol was approved by the University of Stirling Ethics Committee.

### Stimuli – generation and display

The stimuli were generated by a ViSaGe video-graphics card (Cambridge Research Systems Ltd., UK) with 12-bits contrast resolution, presented on a Sony Trinitron monitor, at 120 Hz frame rate and 1024 × 768 spatial resolution. The R (red), G (green) and B (blue) outputs of the monitor were gamma-corrected after calibration with an Optical OP200E photometer. All stimuli were presented in the center of the monitor on a mid-gray background with mean luminance of 65.5 cd/m^2^. Viewing distance was 100 cm.

The multi-color stimuli had a diameter of 12 deg and were made of 96 Gaussian blobs with a standard deviation of 0.08 deg and a Gaussian size standard deviation factor of 5. The stimuli consisted of either two (red, green), three (red, green, blue) or four (red, green, blue, yellow) colors, with respectively 50%, 33% and 25% blobs arranged symmetrically in the symmetric condition, the remaining blobs being randomly positioned and drawn equally from the remaining colors. Example multi-color stimuli are shown in Fig. 2 for two (Fig. 2a), three (Fig. 2b) and four (Fig. 2c) color patterns. For each multi-color pattern, there were four stimulus conditions: (1) the ‘segregated’ condition in which the symmetric blobs were just one color that was the same throughout the session (left panels in Fig. 2); (2) the ‘random-segregated’ condition in which the symmetric blobs were just one color, but the color was randomly selected on each trial; (3) the ‘non-segregated’ condition in which the symmetric blobs were of all colors and in equal proportions (middle panels in Fig. 2), and (4) the ‘anti-symmetric’ condition in which the symmetric blobs were of opposite color across the symmetry axis (right panels in Fig. 2). Each condition was presented 25 times with the four stimulus conditions inter-mixed in random order, resulting in 100 trials within each experimental session. For each number-of-colors condition we ran a minimum of 5 sessions per color-symmetry. For each observer, this resulted in a minimum of 10 sessions for the luminance-defined stimuli, 10 for the two-color (red, green), 15 sessions for three-color (red, green, blue) and 20 sessions for the four-color (red, green, blue, yellow) stimuli, totaling a minimum of 5500 trials per participant. Each observer was measured under two perceptual conditions. First, the observer did not know the symmetry color and no attention-to-color was required. Second, the observer knew beforehand the color of the symmetric blobs in the segregated condition and was required to attend to that color.

**Figure 3:**
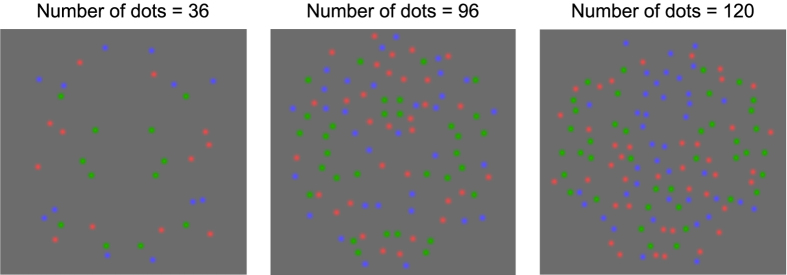
Example three-color symmetric patterns (segregated condition) made of 36 (left), 96 (middle panel) and 120 (right panel) blobs in which 33% blobs arranged symmetrically and the remaining blobs being randomly positioned and drawn equally from the remaining colors. Note that all patterns have the same signal-to-noise ratio.

**Figure 4:**
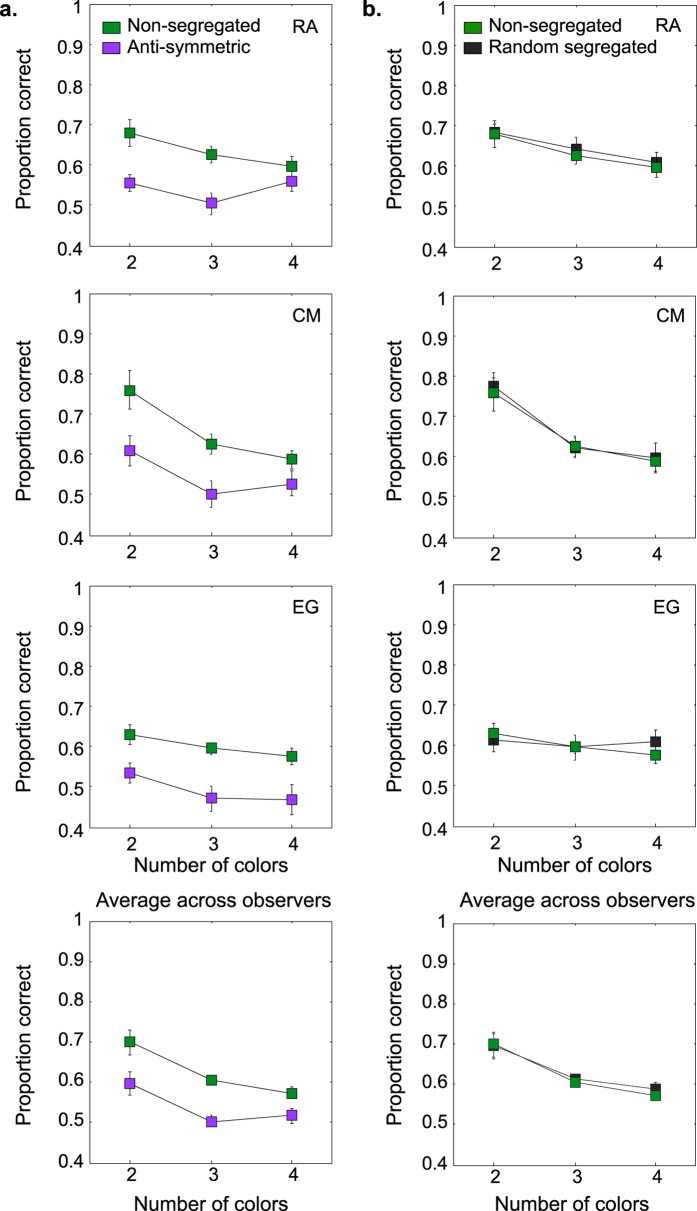
Results with chromatic patterns in the ‘no-attention’ condition are shown for each number of colors. Proportion correct responses for the non-segregated (green symbols) and anti-symmetric (magenta symbols) conditions (**a**) and, for the non-segregated (green symbols) and random-segregated (black symbols) conditions (**b**) are shown for three individual observers and the average across observers.

**Figure 5:**
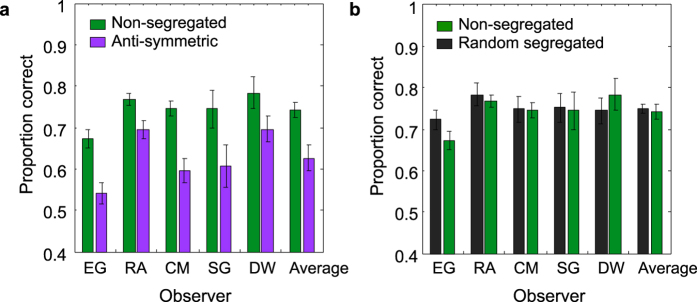
Results with luminance (black and white) patterns in the ‘no-attention’ condition. Proportion correct responses for the non-segregated (green bars) and anti-symmetric (magenta bars) conditions (**a**) and, for the non-segregated (green bars) and random-segregated (black bars) conditions (**b**) are shown for five observers and the average across observers.

**Figure 6:**
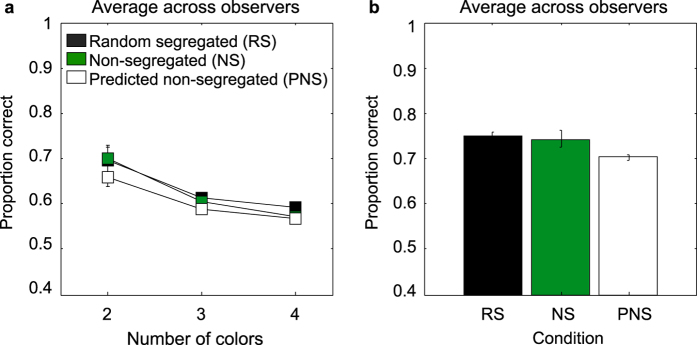
Probability summation of independent color-symmetry channels model predictions for (**a**) chromatic and (**b**) luminance patterns. The average across-observers predicted proportion correct in the non-segregated condition (white symbols) is shown together with the experimental data for random-segregated (black symbols) and non-segregated (green symbols) patterns for each number of colors.

**Figure 7:**
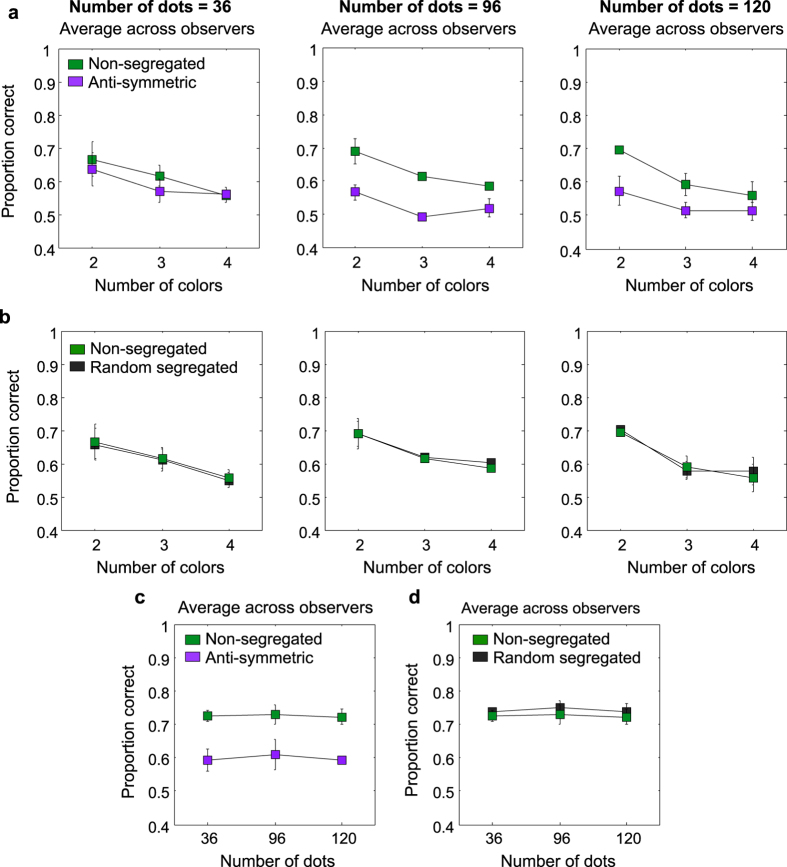
Results with chromatic (**a,b**) and achromatic (**c,d**) patterns made of 36, 96 and 120 blobs in the ‘no-attention’ condition. Average across-observers proportion correct responses for the non-segregated (green symbols) and anti-symmetric (magenta symbols) conditions (**a**) and, for the non-segregated (green symbols) and random-segregated (black symbols) conditions (**b**) are shown for patterns made of 36 blobs (left panels), 96 blobs (middle panels) and 120 blobs (right panels). The individual observers data (three observers) for these conditions are shown in Appendix B, Figures S2 and S3 (*see* Supplementary Information). (**c,d**) Average across-observers proportion correct responses for the achromatic stimuli are shown as a function of number of dots in the stimuli for the non-segregated (green symbols) and anti-symmetric (magenta symbols) conditions (**c**) and the non-segregated (green symbols) and random-segregated (black symbols) conditions (**d**). The individual observers data (three observers) for these conditions are shown in Appendix B, Figure S4 (*see* Supplementary Information).

**Figure 8:**
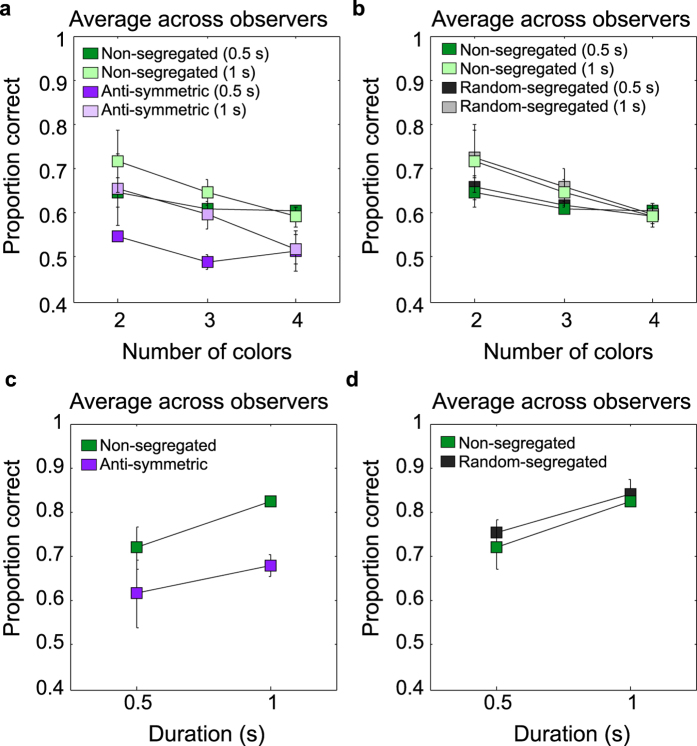
Results for presentation duration 1000 ms. Proportion correct responses (average across two-observers) are shown for the non-segregated (light green symbols) and anti-symmetric (light magenta symbols) conditions (**a**) and, for the non-segregated (light green symbols) and random-segregated (light grey symbols) conditions (**b**). For comparison the results obtained with stimuli presented for 500 ms are also shown (corresponding darker colours). (**c,d**) Average across-observers proportion correct responses for the achromatic stimuli are shown as a function of presentation duration for the non-segregated (light green symbols) and anti-symmetric (light magenta symbols) conditions (**c**) and for the non-segregated (light green symbols) and random-segregated (light grey symbols) conditions (**d**). The individual observers data (two observers) for these conditions are shown in Appendix C, Figure S5 (*see* Supplementary Information).

**Figure 9:**
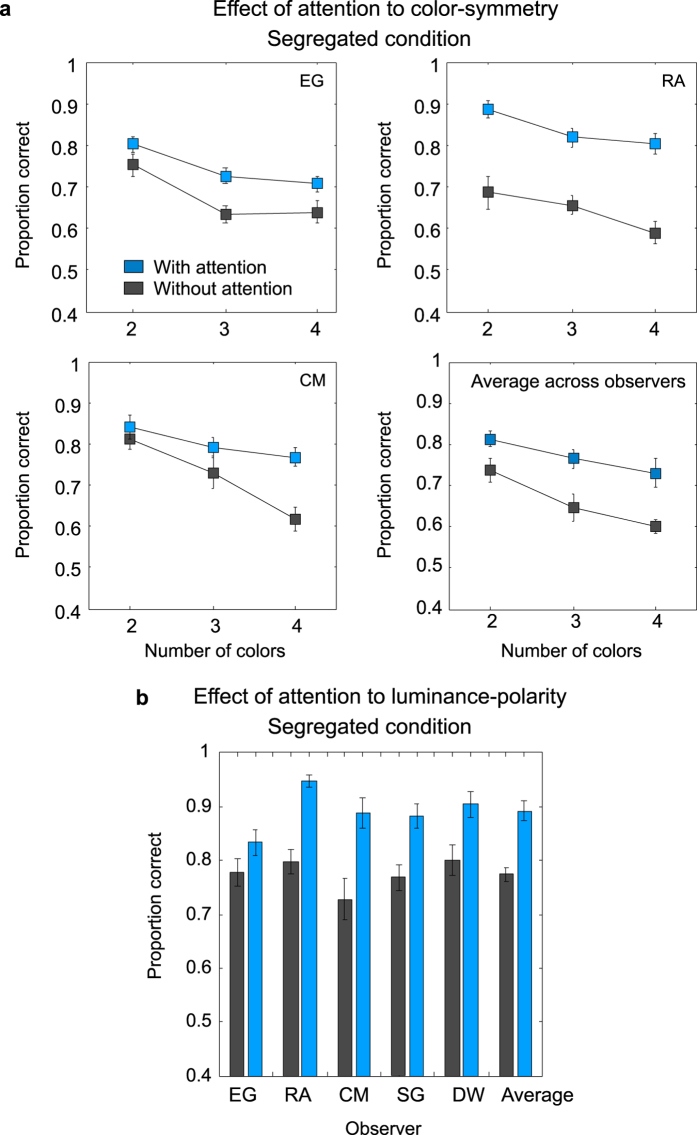
Results for the segregated condition in the ‘with-attention’ (blue symbols) and ‘no-attention’ (gray symbols) conditions with (**a**) chromatic and (**b**) luminance patterns. Proportion correct responses are shown for each number-of-colors condition for three individual observers and the average across observers (**a**). Proportion correct responses are shown for five observers and the average across observers in (**b**).

**Figure 10:**
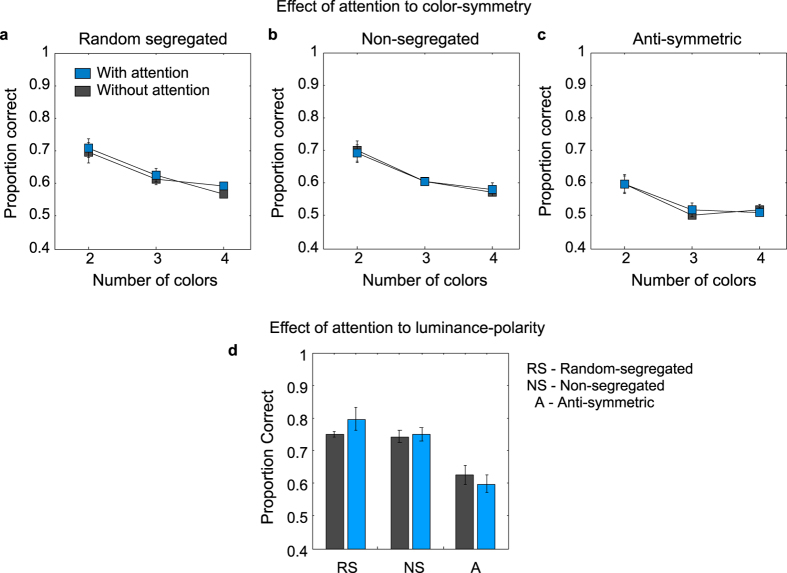
Average across-observers data for the random-segregated, non-segregated and anti-symmetric (**a**) chromatic and (**b**) luminance conditions obtained in the ‘with-attention’ (blue symbols) and ‘no-attention’ (gray symbols) conditions.

In two subsequent experiments we tested all the aforementioned without-attention conditions and examined whether the number of blobs in the stimulus (or blob density) and stimulus presentation duration (1000 ms) affected symmetry detection. For the number of blobs experiment we used patterns made of 36 and 120 blobs. The higher number of 120 blobs was limited by the stimulus area in which blobs could be drawn without overlap. Figure 3 shows example three-color patterns made of different number of blobs: 36 (left panel), 96 (middle panel) and 120 (right panel). The reader should note that the symmetry signal-to-noise ratio is identical (i.e. 33%) in all these patterns.

For the additional presentation duration experiment, each stimulus was presented for 1000 ms. We used patterns made of 96 blobs and each condition was presented 25 times with the four stimulus conditions inter-mixed in random order, resulting in 100 trials within each experimental session.

### Procedure – measurement of isoluminant points and contrast matching

For our multi-color stimuli we wanted to ensure that the red, green, blue and yellow patterns were isoluminant. Isoluminance was measured using the criterion of minimum flicker^42^. First, to determine the isoluminant point of a fully-saturated blue, we used a stimulus comprising of a quasi-random pattern of blue-blobs on the average luminance (gray) background (same background was used in all later experiments). The blob-pattern was changed at about 2.0 Hz. The contrast of the blue blobs was set to maximum. Participants adjusted the luminance of the blue Gaussian blobs from a random starting point until the perceived flicker stopped or was at a minimum. There was no time limit for the isoluminance setting procedure. Each subject made 10 settings per session from which a mean value was estimated and, the process repeated for five sessions across different days. We calculated the blue isoluminant measure from all these measurements. These are shown in *Appendix D* (*see* Supplementary information) for each observer.

To determine the isoluminant point for red, we used two-color stimuli made of blue and red blobs. For each subject, the contrast of the blue blobs was fixed to 1 and the blue isoluminant point was set to the value determined in the first experiment described above. Participants adjusted the luminance of the red blob patterns from a random start point until the perceived flicker stopped or was at a minimum. Each subject made 10 settings per session, and for three sessions repeated on different days. We calculated the red isoluminant measure from all these five measurements.

After the red isoluminant point was measured, a contrast matching experiment was carried out to equate the perceived contrast of the blue and red blobs. The matched contrast was determined using an ongoing stimulus presentation in which the blue blobs were fixed, at maximum contrast and the red-blobs contrast was adjustable. Participants used the keys on the response box to adjust the contrast of the red-blobs until they matched the perceived contrast of the blue-blobs in the pattern. There was no time limit for the contrast matching procedure. Each subject made 10 settings from which the mean value was estimated. This was repeated five times on different days, and the mean value of red contrast was calculated from these measurements.

Using stimuli made of blue and green blobs we used a similar procedure to determine the green isoluminant point and green contrast. Similarly, using stimuli made of blue and yellow blobs we determined the yellow isoluminant point and yellow contrast. The red, green, blue and yellow isoluminant points and matched contrasts are shown in *Appendix D* for each individual observer (*see* Supplementary information).

### Procedure – 2IFC

To measure symmetry detection we used a two-interval-forced-choice (2IFC) procedure. On each trial a stimulus corresponding to one of the four conditions, i.e. either ‘segregated’, ‘random segregated’, ‘non-segregated’ or ‘anti-symmetric’ was randomly presented in one of the two intervals while the other interval (i.e. the null interval) contained a stimulus made of quasi-randomly positioned blobs with equal numbers of the relevant colors. Each stimulus was presented for 500 ms with an inter-stimulus interval (ISI) of 500 ms. A tone indicated the beginning of each interval. Trials were initiated by the previous key press, with an interval of 500 ms before the onset of the first stimulus. The task for the subject was to indicate by a key press the interval containing the symmetric stimulus. It was explained to the subject that the symmetry of the pattern was around the vertical axis. Each of the four stimulus conditions (i.e. segregated, random segregated, non-segregated and anti-symmetric) was presented 25 times, in random order. This resulted in 100 trials per session. For each stimulus condition, we measured proportion correct answers. The experiment was blocked by the number of colors in the stimulus, i.e. two-color, three-color and four-color stimuli, in order to reduce uncertainty.

In addition, we ran different sessions in which the segregated pattern was one of the available colors. For each color-symmetry condition we ran a minimum of 5 sessions. For each observer, this resulted in a minimum of 10 sessions for the luminance-defined stimuli (5 white-symmetry and 5 dark-symmetry) and two-color stimuli (5 red-symmetry and 5 green-symmetry) stimuli. We then calculated the average proportion correct answers and standard error of these differences across the 10 sessions. These means and standard errors are the ones shown on the graphs.

For the three-color stimuli there were 15 sessions (5 red-symmetry, 5 green-symmetry and 5 blue-symmetry) and for the four-color stimuli there were 20 sessions (5 sessions per color). The average proportion correct detections and standard error of these differences across these sessions are shown in all the graphs.

Each observer was measured under two perceptual conditions. First, the observer did not know a-priori the symmetry color and no attention-to-color was required. Thus, this perceptual condition was carried out first for all observers. Second, the observer knew beforehand the color of the symmetric blobs in the segregated condition and was required to attend to that color.

## Additional Information

**How to cite this article**: Gheorghiu, E. *et al*. The role of color and attention-to-color in mirror-symmetry perception. *Sci. Rep.* **6**, 29287; doi: 10.1038/srep29287 (2016).

## Supplementary Material

Supplementary Information

## Figures and Tables

**Figure 1:**
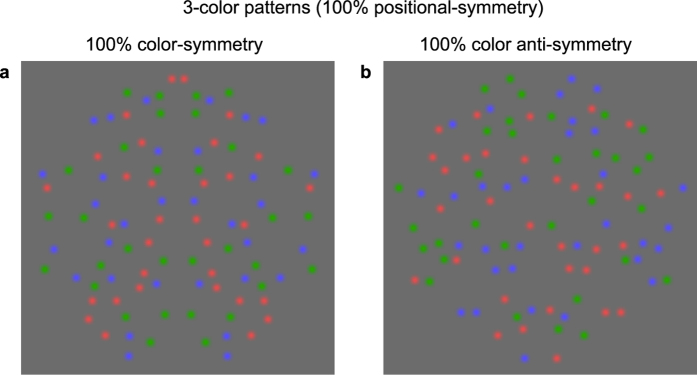
(**a**) Pattern with perfect positional and color symmetry. Note that the symmetry is perceived effortlessly. (**b**) Pattern with perfect positional symmetry, but with the colors mismatched across the symmetry axis. This time the symmetry is hard to perceive.
