# Supplementary material for: The role of color and attention-to-color in mirror-symmetry perception
Source: Sci Rep. 2016 Jul 11;6:29287. doi: 10.1038/srep29287 (PMC4941524; doi:10.1038/srep29287)
Supplement: Supplementary Information [file srep29287-s1.pdf]

Supplementary Information

The role of color and attention-to-color in mirror-symmetry perception

Elena Gheorghiu[1], Frederick AA Kingdom[2], Aaron Remkes[1], Hyung-Chul O Li[3] & Stéphane Rainville[4]

[1] University of Stirling, Department of Psychology, Stirling, FK9 4LA, Scotland, United Kingdom
[2] McGill Vision Research, Department of Ophthalmology, McGill University, Montreal, Canada
[3] Kwangwoon University, Department of Industrial Psychology, Seoul, Korea
[4] VizirLabs Consulting, Chelsea, Quebec, Canada

Appendix A: Probability summation model equation

To test the model we used the equation for the probability summation of equal intensity stimuli given in Kingdom et al. [16], as follows:

$$Pc = n\int_{-\infty}^{\infty}\phi\left(t-d'\right)\Phi\left(t\right)^{QM-n}\Phi\left(t-d'\right)^{n-1}dt + \ldots. + \left(Q-n\right)\int_{-\infty}^{\infty}\phi\left(t\right)\Phi\left(t\right)^{QM-n-1}\Phi\left(t-d'\right)^{n}dt$$

where $d' = \left(gs\right)^{\tau}$

Figure S1 illustrates some of the parameters.

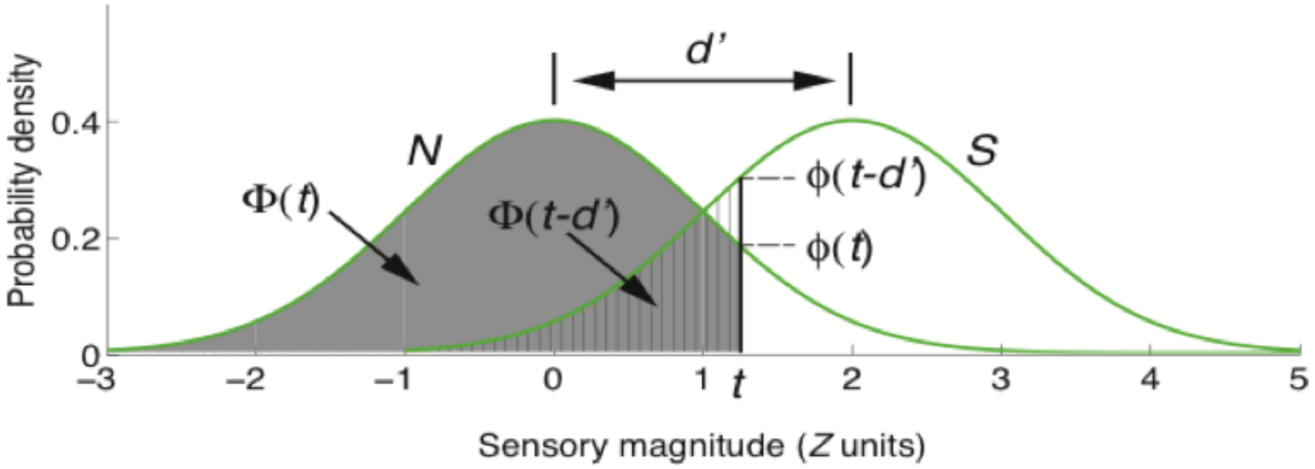

Figure S1. Parameters for calculating probability sumamtion under the assumptions of Signal Detection Theory. *N* – noise distribution; *S* – stimulus (or signal) distribution; *t* – sample sensory magnitude; *d*' – separation between noise and signal distributions; $\Phi(t)$ and $\Phi(t\text{-}d')$ are the areas under the noise and signal distributions to the left of *t*; $\phi(t)$ and $\phi\,(t\text{-}d')$ are the heights of the noise and signal distributions at *t*. Based on Figure 3 in Kingdom et al. [16].

As illustrated in Fig.S1, *t* is sample stimulus strength, $\phi(t)$ and $\phi(t\text{-}d')$ are the heights of the noise (*N*) and signal (*S*) distributions at *t*, $\Phi(t)$ and $\Phi(t\text{-}d')$ are the areas under the noise and signal distributions to the left of *t*. In the equation itself, *s* is stimulus strength, *g* is a scaling factor to translate stimulus strength into the signal detection parameter *d*', τ is the exponent of the power function that translates external to

internal response strength, n is the number of channels containing a stimulus, Q the number of monitored channels and M the number of alternatives in the forced-choice task (here 2). Full expositions of the equation are provided in Kingdom et al. [16].

Appendix B: Effect of number of blobs on symmetry detection

Proportion correct responses for three individual observers as well as the average across-observers for the non-segregated and anti-symmetric conditions are shown in Fig.S2 and, for the non-segregated and random-segregated conditions are shown in Fig.S3. The results obtained with the patterns made of 36 blobs are shown in the left panels, for 96 blobs in the middle panels and 120 blobs in the right panels. The corresponding results for the luminance-polarity (bright and dark blobs) are shown in Fig.S4a-b.

For each number of blobs condition, the logit transformed values of the data shown in Fig.S2 were submitted to a repeated measure ANOVA with factors Stimulus condition (anti-symmetric vs. non-segregated) and number of color condition (2, 3 and 4 colors). There was a statistically significant effect of Stimulus condition for all number of blobs conditions at 0.05 level ($F(1,6)=6.433$, $p=0.044$ for 36 blobs; $F(1,6)=36.52$, $p=0.0009$ for 96 blobs, and $F(1,6)=9.569$, $p=0.021$ for 120 blobs). The effect of number of colors was also found to be significant ($F(1,6)=5.923$, $p=0.038$ for 96 blobs, and $F(1,6)=6.605$, $p=0.031$ for 120 blobs) except for 36 blob density ($F(1,6)=1.703$, $p=0.256$). There were no statistically significant interactions between stimulus condition and number of colors.

Similar statistical analysis was carried out for the non-segregated and random-segregated conditions shown in Fig.S3. For each number of blobs condition, the logit transformed data were submitted to a repeated measure ANOVA with factors Stimulus condition (random-segregated vs. non-segregated) and number of color condition (2, 3 and 4 colors). There was no statistically significant effect of Stimulus condition for all number of blobs conditions at 0.05 level ($F(1,6)=1.254$, $p=0.306$ for 36 blobs; $F(1,6)=0.967$, $p=0.363$ for 96 blobs, and $F(1,6)=1.726$, $p=0.237$ for 120 blobs). The effect of number of colors was also found not statistically significant ($F(1,6)=2.106$, $p=0.203$ for 36 blobs; $F(1,6)=3.487$, $p=0.099$ for 96 blobs) except for 120 blob density ($F(1,6) = 6.828$, $p = 0.284$). There were no statistically significant interactions between stimulus conditions and number of colors.

For the achromatic conditions shown in Fig.S4a, the logit transformed data were submitted to a repeated measure ANOVA with factors Stimulus condition (anti-symmetric vs. non-segregated) and blob density (36, 96 and 120 blobs). There was a statistically significant effect of Stimulus condition (anti-symmetric vs. non-segregated) ($F(1,6)=162.3$, $p<0.0001$) and no significant effect of blob density ($F(1,6)=0.064$, $p=0.938$. For the data shown in Fig.4Sb, there were no significant effects of Stimulus

condition (non-segregated vs. random-segregated) (F(1,6)=2.633, p=0.156) and blob density (F(1,6)=0.1345, p=0.877) and no significant interactions.

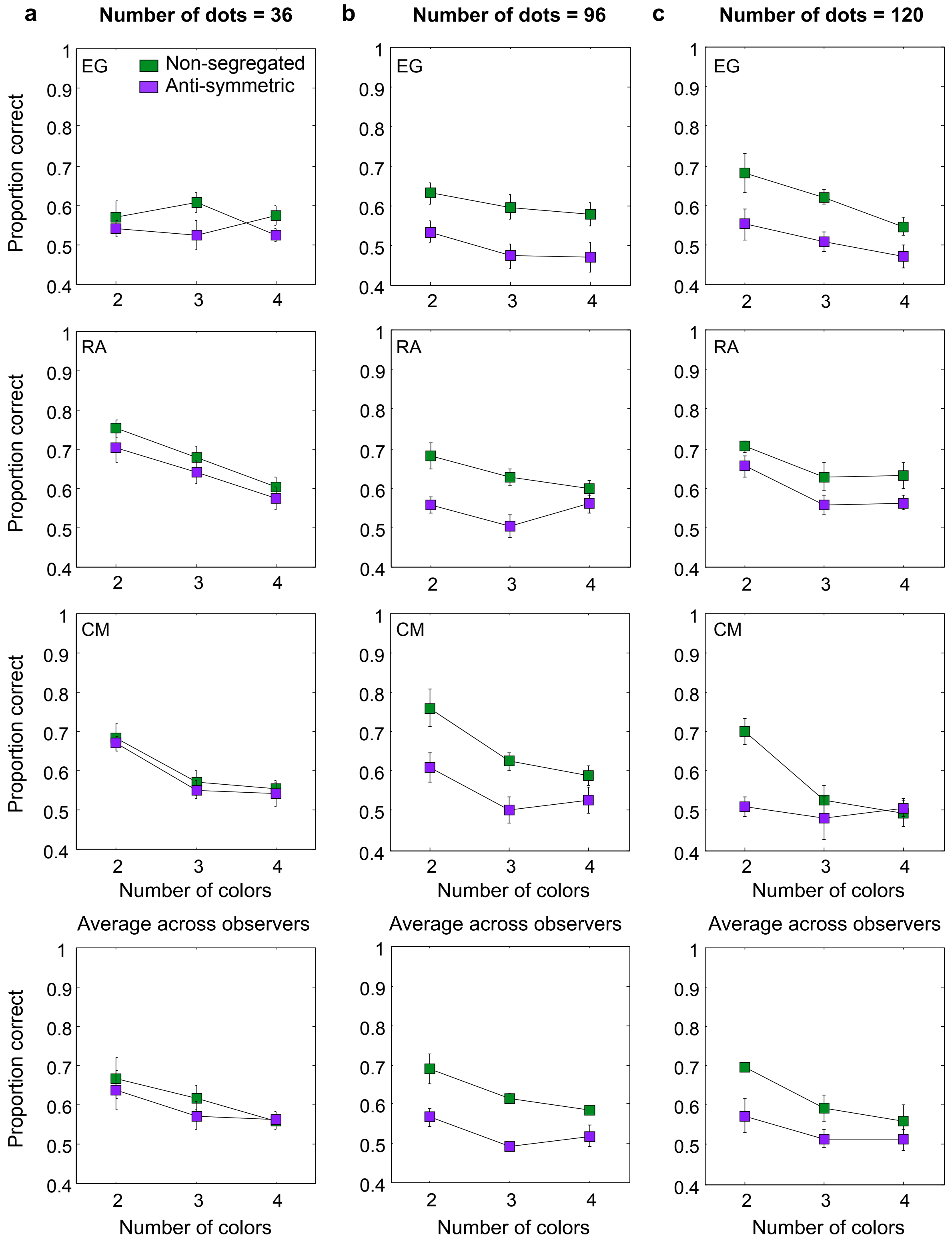

Figure S2. Individual observers and average across-observers proportion correct responses for the non-segregated (green symbols) and anti-symmetric (magenta symbols) conditions obtained with chromatic patterns made of (a) 36 blobs, (b) 96 blobs and (c) 120 blobs in the 'no-attention' condition.

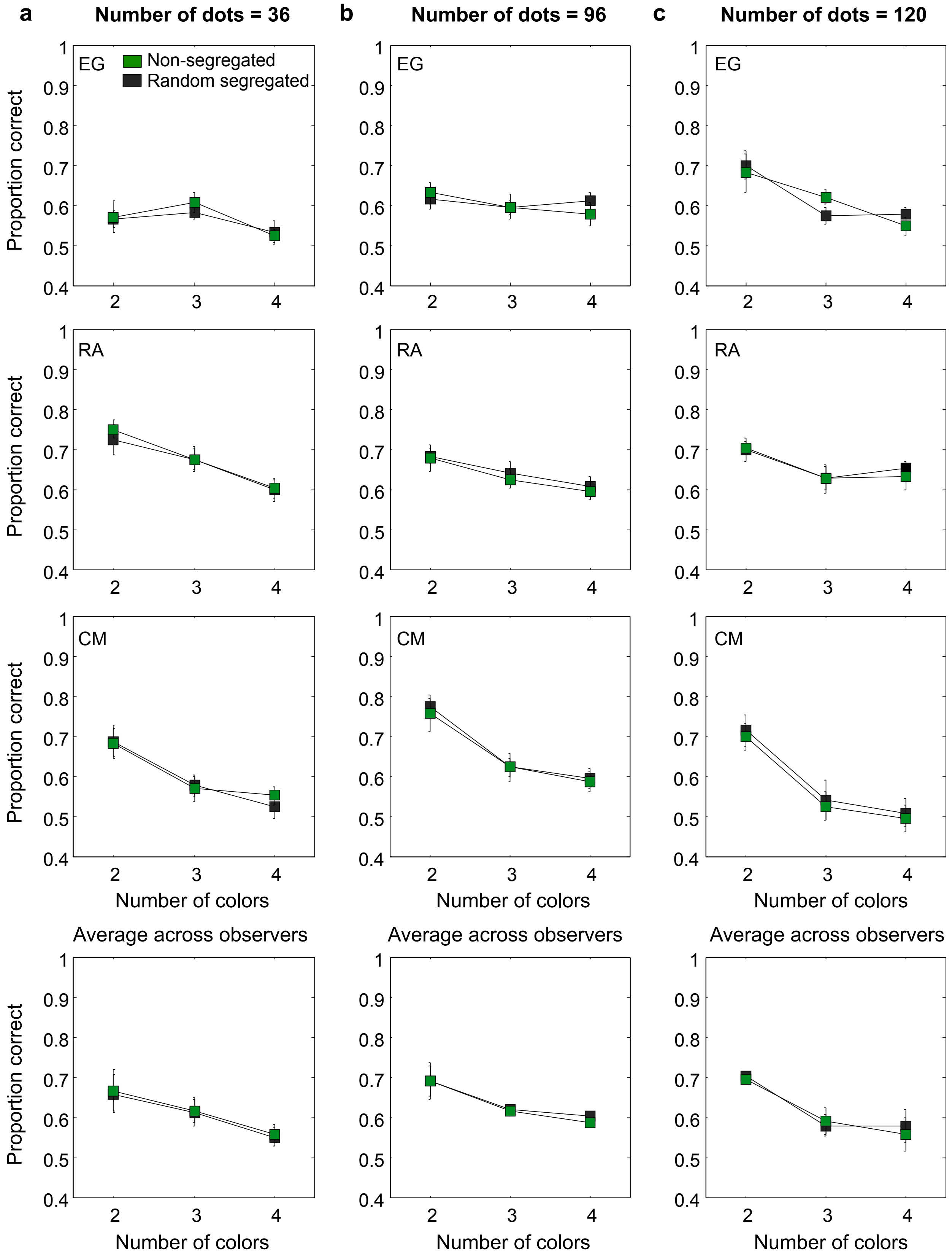

Figure S3. Individual observers and average across-observers proportion correct responses for the non-segregated (green symbols) and random-segregated (black symbols) conditions obtained with chromatic patterns made of (a) 36 blobs, (b) 96 blobs and (c) 120 blobs in the 'no-attention' condition.

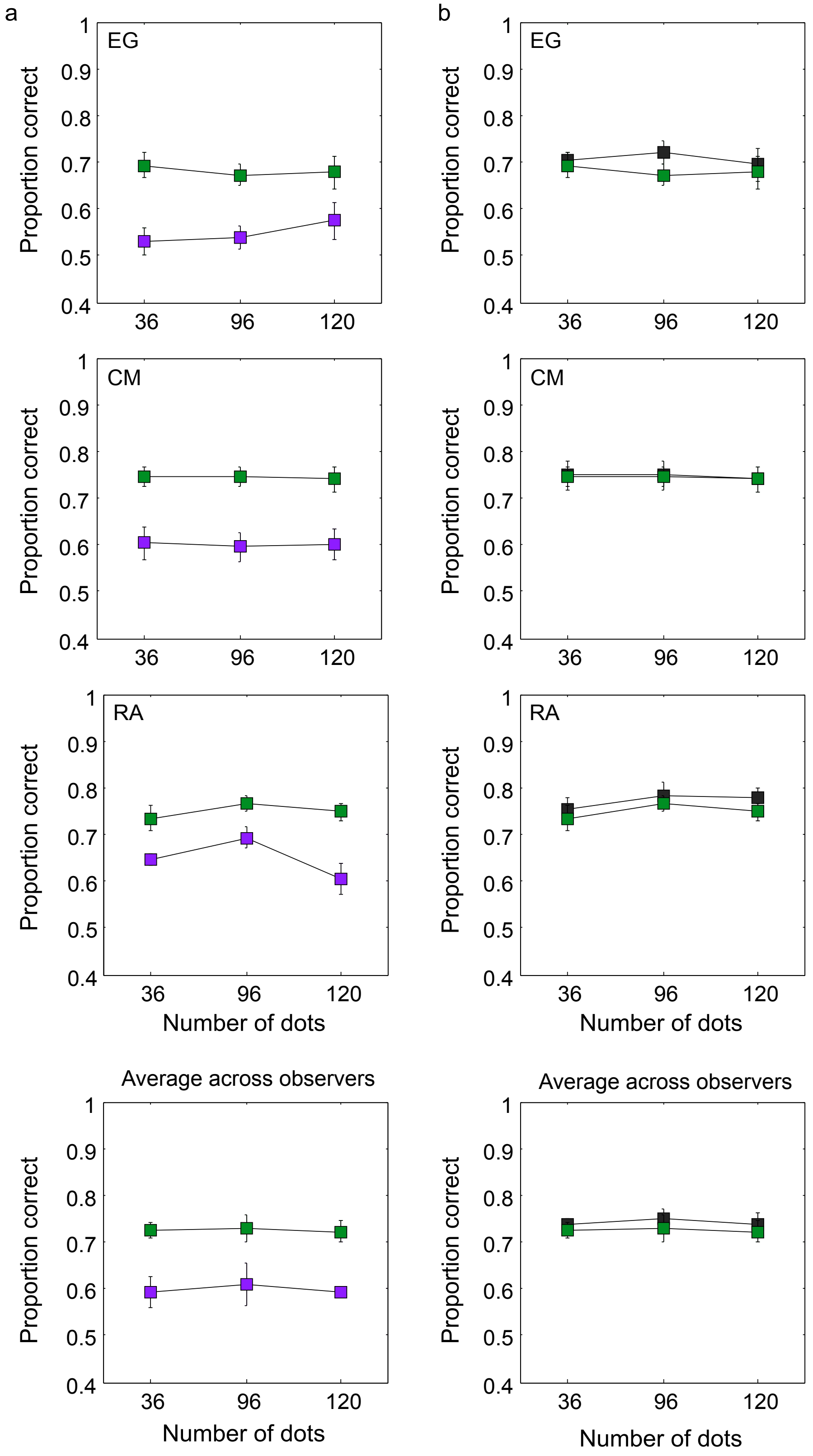

Figure S4. Individual observers and average across-observers proportion correct responses for the achromatic stimuli are shown as a function of number of blobs in the stimuli for the non-segregated (green symbols) and anti-symmetric (magenta symbols) conditions (a) and the non-segregated (green symbols) and random-segregated (black symbols) conditions (b).

Appendix C: Effect of stimulus duration on symmetry detection

Figure S5 presents results for both 0.5 and 1 sec durations for two observers and the average across observers. Proportion correct results are shown in Fig.S5a for the non-segregated (light green symbols) and anti-symmetric (light magenta symbols) conditions and in Fig.S5b for the non-segregated and random-segregated (light grey symbols) conditions. For comparison reason we also show the results obtained with 500 ms presentation duration (darker symbols). The corresponding results for the luminance-polarity are shown in Fig.S5c-d. These results show that doubling the presentation duration to 1 sec produces only a modest increase in performance (compare light and dark magenta/green/black symbols), that is, ~0.75 for the two-color and 0.85 proportion correct for the achromatic conditions.

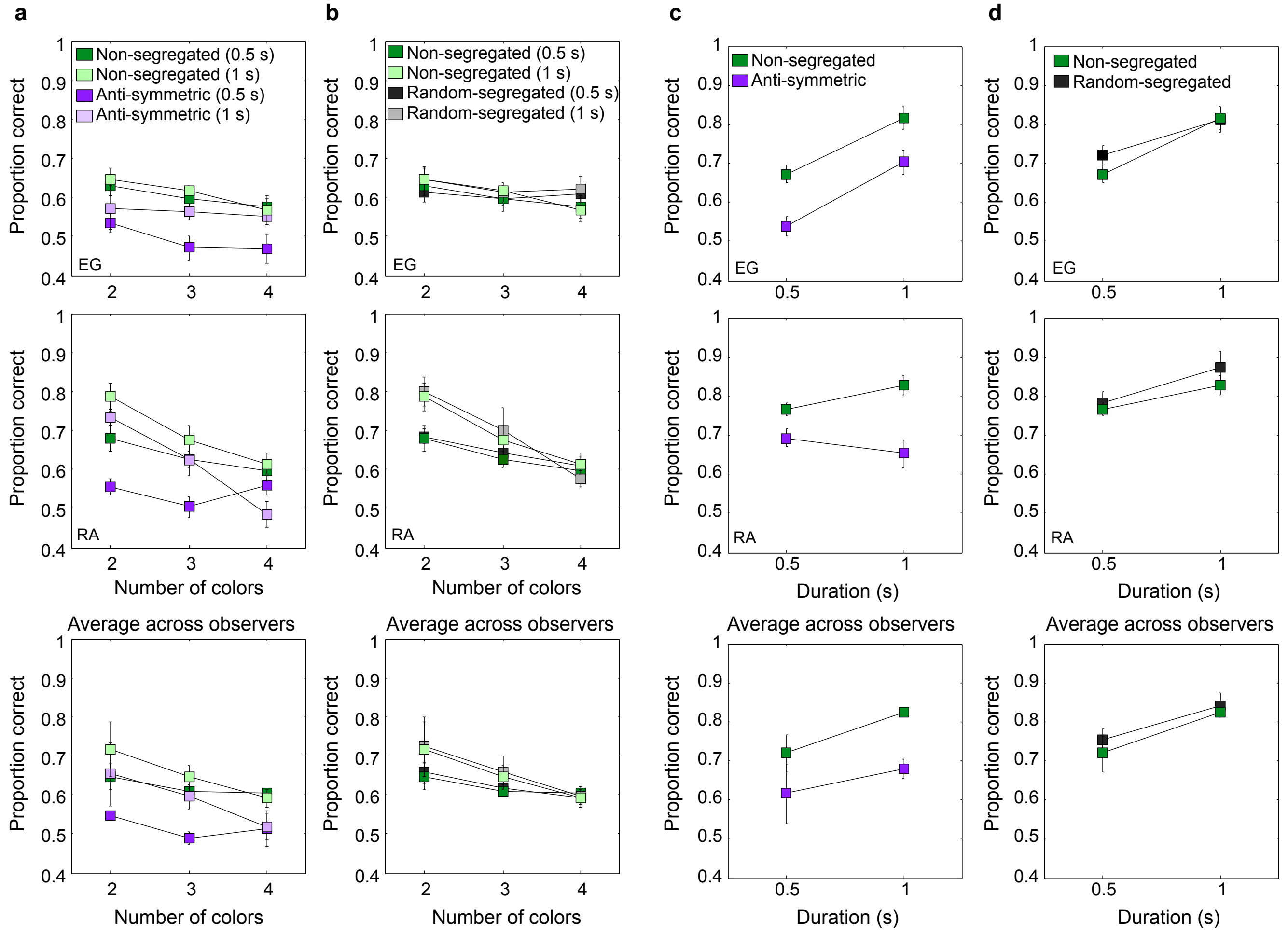

Figure S5. Individual observers and average across-observers results for presentation duration of 1000 ms. Proportion correct responses for the non-segregated (light green symbols) and anti-symmetric (light magenta symbols) conditions (a) and, for the non-segregated (light green symbols) and random-segregated (light grey symbols) conditions (b). For comparison reason the results obtained with stimuli presented for 500 ms are also shown (corresponding darker colours). (c-d) Proportion correct responses for the achromatic stimuli are shown as a function of presentation duration for the non-segregated (light green symbols) and anti-symmetric (light magenta symbols) conditions (c) and the non-segregated (light green symbols) and random-segregated (light grey symbols) conditions (d).

Appendix D: Isoluminant points and matched contrasts

The red, green, blue and yellow isoluminant points and matched contrasts for each individual observer (see *Methods* section for details).

Subject	Blue		Red		Green		Yellow	
	Isoluminant Ratio	Contrast	Isoluminant Ratio	Contrast	Isoluminant Ratio	Contrast	Isoluminant Ratio	Contrast
EG	0.102	1	0.272	0.585	1.883	0.3370	5.6940	0.1365
RA	0.158	1	0.246	0.898	1.809	0.4235	4.9050	0.1850
CM	0.144	1	0.253	0.869	1.858	0.4667	5.7453	0.1583
JS	0.182	1	0.452	0.549	1.647	0.4090	5.549	0.133
DW	-	-	0.193	0.700	2.355	0.273	-	-
SG	-	-	0.210	0.700	2.469	0.277	-	-
